# Effects of past mating behavior versus past ejaculation on male mate choice and male attractiveness

**DOI:** 10.1093/beheco/arae002

**Published:** 2024-01-17

**Authors:** Meng-Han Joseph Chung, Megan L Head, Rebecca J Fox, Michael D Jennions

**Affiliations:** Division of Ecology and Evolution, Research School of Biology, Australian National University, 46 Sullivans Creek Road, Acton, Australian Capital Territory 2600, Australia; Division of Ecology and Evolution, Research School of Biology, Australian National University, 46 Sullivans Creek Road, Acton, Australian Capital Territory 2600, Australia; Division of Ecology and Evolution, Research School of Biology, Australian National University, 46 Sullivans Creek Road, Acton, Australian Capital Territory 2600, Australia; Division of Ecology and Evolution, Research School of Biology, Australian National University, 46 Sullivans Creek Road, Acton, Australian Capital Territory 2600, Australia; Stellenbosch Institute for Advanced Study (STIAS), Wallenberg Centre, 10 Marais Street, Stellenbosch 7600, South Africa

**Keywords:** male mate choice, mating history, mating behavior, mating experience, reproductive cost, sperm competition

## Abstract

Past reproductive effort allows males to assess their ability to acquire mates, but it also consumes resources that can reduce their future competitive ability. Few studies have examined how a male’s reproductive history affects his subsequent mate choice, and, to date, no study has determined the relative contribution of past mating behavior and past ejaculate production because these two forms of investment are naturally highly correlated. Here, we disentangled the relative effects of past mating behavior and past ejaculate production in mosquitofish (*Gambusia holbrooki*) by experimentally preventing some males from ejaculating when trying to mate. We assessed the effect of mating behavior on mate choice by comparing males that had previously been with or without access to females and male rivals for 8 and 16 weeks and assessed the effect of ejaculation on mate choice by comparing males that either could or could not ejaculate when they had access to females for 16 weeks. Reproductive treatment did not affect male attractiveness, but it did affect male mate choice. Somewhat surprisingly, in five of the six treatment-by-age at testing combinations, males preferred a female in the vicinity of a male rival over a solitary female. This preference was marginally stronger for males that had previously engaged in mating behavior but were unaffected by past ejaculate production. We discuss the potential benefits to males of associating with another male when seeking mates. This is the first study to quantify the relative influence of pre- and post-copulatory reproductive investment on male mate choice.

## Teaser

When choice is cheap, males should mate selectively, and a male’s reproductive history could affect his mating preferences. Experience increases males’ ability to estimate their mating potential but might lower their sexual competitiveness due to past reproductive investment. Here, we separate the effects of past mating behavior and ejaculation on male mate choice. We found that males preferred females near another male and that past mating behavior, but not ejaculation, tended to increase this preference.

## INTRODUCTION

Mate choice is the process by which individuals select preferred mates, resulting in non-random patterns of copulation and/or fertilization. Most research is on female rather than male mate choice due to the lower costs of mate rejection (i.e., choosiness) for females. The costs are lower because of the greater availability of potential mates for females due to the shorter “time out” of males after mating (Andersson and Simmons 2006; Jennions and Kokko 2013). Expensive reproductive investment by males (e.g., Berger-Tal and Lubin 2011; Cady et al. 2011; Boivin 2013) can, however, increase the “time out” of males and thereby favor the evolution of male mating preferences that involve the rejection of lower quality females (Edward and Chapman 2011). High costs of reproduction for males can also favor the strategic allocation of male mating effort and/or ejaculates. For example, male *Drosophila melanogaster* with costly sperm production exhibit shorter copulations and transfer fewer sperm when mating with small, less fecund females (Lüpold et al. 2011).

Reproductive history is thought to play a crucial role in shaping context-dependent mate choice. Individuals can learn from previous encounters with potential mates and better assess both their own attractiveness and mate availability (Fawcett and Bleay 2009), hence estimating the costs of rejecting a potential mate. Empirical studies consistently demonstrate that naïve (i.e., virgin) and previously mated individuals behave differently when they have the opportunity to mate (Balaban-Feld and Valone 2018; Daimon et al. 2022; but see Dougherty 2021). Naïve males are sometimes less attractive to females (Edvardsson et al. 2008; Iyengar 2009; Milonas et al. 2011; Kong et al. 2021) and/or are less capable of distinguishing among potential mates (Hebets and Sullivan-Beckers 2010). Both factors should reduce the choosiness of naïve males (Edward and Chapman 2011).

Reproductive history can also affect the choosiness of males due to associated costs. In species with intense competition for females, males often increase their reproductive effort, leading to physical injury and resource depletion (Emery Thompson and Georgiev 2014). These costs can lower a male’s future performance and elevate the onset of senescence (Lemaître et al. 2015). On the one hand, these costs might reduce future male attractiveness (Muller et al. 2016a,b) and/or competitive ability (Koppik et al. 2018; Hopkins et al. 2019), which increase the costs of choosiness (i.e., mate rejection has a greater effect on a male’s actual mating rate). On the other hand, lower residual reproductive value due to fewer future mating opportunities (Macartney et al. 2020) might trigger more prudent mate choice (Härdling and Kokko 2005) when a male’s reproductive budget is constrained (Dewsbury 1982). Reproductive history could, therefore, have a mixed effect on male mate choice: benefits of information acquisition and costs of past reproductive effort. The influence of reproductive history on female mate choice has received much attention (meta-analysis: Richardson and Zuk 2023), but it remains unclear how past reproductive effort affects male mate choice. Furthermore, almost nothing is known about how different components of male reproductive history affect male mate choice. To reproduce, males must invest into both mating effort (e.g., courtship, fighting) and ejaculate production (e.g., nuptial gifts, sperm quality). Both aspects of a male’s reproductive history might affect his subsequent mate choice.

Past investment in mating effort by males can convey beneficial information about attractiveness or competitiveness and the likelihood of sperm competition. For example, the outcome of male–male contests can create “winner-loser” effects, whereby past winners adjust their behavior and have higher subsequent reproductive success (Trannoy et al. 2016; Filice and Dukas 2019; Kola et al. 2021; review: Hsu et al. 2006). In field crickets and wolf spiders, prior exposure to male courtship enhances female choosiness (Hebets and Vink 2007; Atwell and Wagner Jr 2014) and alters how much effort males put into mating (Clark et al. 2012, 2015; Gray and Simmons 2013). Similarly, past ejaculate investment (e.g., sperm release) can reinforce subsequent sexual behavior (Tenk et al. 2009) and strengthen the mating preferences of male rats (Kippin and Pfaus 2001).

Past investment in mating effort by males can also be energetically expensive and impose costs (Somjee et al. 2018; O’Brien et al. 2019) that lower body condition (Mappes et al. 1996), reduce endurance (Rometsch et al. 2021) and thereby affect future reproductive success. For example, male *D. melanogaster* who had invested into mating behavior (controlling for the rate of ejaculation) die sooner than naïve males (Cordts and Partridge 1996). This decline in residual reproductive value is likely to affect male choosiness. Similarly, investment into ejaculates may impose costs that affect male mate choice (Lemaître et al. 2020). Males that ejaculate frequently experience faster sperm depletion (Montrose et al. 2004; Schütz et al. 2017; Abe 2019) that can lead them to selectively allocate ejaculates to different females (e.g., Hunter et al. 2000).

To date, the *relative* contributions of past mating behavior and past ejaculate investment to future male mate choice has been understudied. This is due to the tight association between these two forms of investment: males that ejaculate and replenish sperm have already put effort into mating. This strong correlation makes it challenging to test whether shifts in male mating preferences are due to their past mating behavior, past ejaculate production, or both. To conduct these tests requires experiments, or unusual natural histories, that break the natural correlation. A handful of studies have quantified the separate effects of pre- and post-copulatory investment by males on their future performance (Cordts and Partridge 1996; Olsson et al. 1997; Chung et al. 2021), but only one study has measured the effects on male mate choice. Chung et al. (2021) ran an experiment to show that male mosquitofish (*Gambusia holbrooki*) who had experienced *both* mating behavior and ejaculation had a weaker tendency to associate with large females but that neither past mating behavior nor past ejaculate investment on their own affected male mate choice. One shortcoming of this experiment is that it was conducted for a relatively short period, and focal males were not exposed to rivals. This is unfortunate as physical combat and a greater perceived risk of sperm competition due to the presence of rivals are key elements in male reproductive history, shaping subsequent mating decision (e.g., actively avoiding conflict; Hsu et al. 2006; Plath et al. 2008).

Here, we investigate the relative effects of pre- and post-copulatory investment on mate choice by male mosquitofish (*G. holbrooki*) under competitive conditions. Males exclusively adopt a coercive mating strategy: they approach females from behind and, when close enough, swing their gonopodium (intromittent organ) forward to insert the tip into the female’s genital tract and expel sperm bundles. Males persistently attempt to mate (up to two attempts per minute; Iglesias-Carrasco et al. 2019), and previous studies have documented a male preference for novel females (Vega-Trejo et al. 2014) or larger females (Callander et al. 2012), while females tend to associate with larger males (Bisazza et al. 2001) or those in better body condition (Kahn et al. 2012). There is also evidence of winner–loser effects, where victorious males have greater access to females and higher copulation success (Harrison et al. 2018, 2023). Males face high levels of sperm competition: females typically mate multiply and can store sperm for up to six months (Evans and Pilastro 2011). Ejaculate production is costly and covaries with male body size, diet, and age (O’Dea et al. 2014; Aich et al. 2021).

To tease apart the effects of mating effort and ejaculate investment, we have developed an ablation technique to remove the tip of the male’s gonopodium. Tip removal prevents males from sperm release, but otherwise has no effects on male behavior, sperm production, or attractiveness (Chung et al. 2019, 2020, 2021; Fox et al. 2019). Therefore, ablated males do not ejaculate when they attempt to copulate, which lowers the rate of sperm replenishment. In this study, we quantified the effect of mating effort by comparing naïve males that were isolated from other individuals (i.e., no sexual experience) to ablated males that could freely interact with a female and two rivals (i.e., experiencing mating behavior but not ejaculation); and we quantified the effect of ejaculation by comparing ablated and non-ablated (i.e., natural state) males that could interact with a female and two rivals (i.e., experiencing both mating behavior and ejaculation). We evaluated the cumulative effect of past reproductive effort by conducting male mate choice trials twice: after 8 and 16 weeks in the treatments. These correspond to the midpoint and the end of the breeding season in the wild population (Kahn et al. 2013).

## MATERIALS AND METHODS

### Origin and maintenance of fish

We collected eastern mosquitofish (*Gambusia holbrooki*) from ponds in Canberra, Australia in May 2020. Adult fish were transferred into single-sex 90L stock tanks (40–50 fish/tank) and used as stimulus (non-focal) individuals. Juveniles were raised in mixed-sex groups in stock tanks. We inspected them to determine their sex twice a week. Immature males were identified based on their elongated anal fin and transferred into single-sex stock tanks before reaching maturity (i.e., before the gonopodium was fully developed with visible claws). Fish were kept at 28 ± 1 °C under 14:10 light:dark cycle and fed twice daily. We used commercial fish flakes for fish in stock tanks and *Artemia* nauplii ad libitum for focal fish in individual tanks. The experiment was run from August 2020 to January 2021.

### Experimental design

To identify sexually active males, we randomly selected a virgin male and placed him in a 4L tank with a wild-caught female for 5 min. Only males that actively attempted to insert their gonopodium tip into the female’s gonopore (indicating a mating attempt) were used as focal males. Focal males were then anesthetized in an ice slurry for 10 s prior to photographing. Standard length (SL: snout tip to base of caudal fin) was later measured from the photographs using *ImageJ* (Abràmoff et al. 2004). Following a recovery period of at least seven days, focal males (range: 17.9–27.4 mm SL) were randomly assigned to one of the three treatments (*n* = 60 per treatment). There was no size difference among treatments (*F*_2,177_ = 1.896; *P *= 0.153):

(a) “Naïve”: a non-ablated, virgin male (i.e., intact gonopodium tip), a wild-caught female, and two wild-caught rival males were placed in three separate compartments of a 7L tank using mesh barriers. The focal male experienced olfactory and visual cues from the female and male rivals, but had no physical contact with the female or male, or the opportunity to ejaculate while mating.
(b) “Mating only”: an ablated, virgin male had the tip of his gonopodium removed (see below). This prevents males from receiving mechano-sensory stimuli that induce ejaculation (Chung et al. 2020). The male was placed with a wild-caught female and two male rivals in a 7L tank. He could freely interact with the rivals and attempt to mate with the female, but he was unable to ejaculate. The males’ investment in reproduction was thus primarily into pre-copulatory behavior.
(c) “Mating and ejaculation”: a non-ablated (i.e., natural state), virgin male was placed with a female and two wild-caught males in a 7L tank. The focal male could freely interact with the rivals, attempt to mate with the female and could also ejaculate. These focal males invested into both pre-copulatory mating behavior and post-copulatory ejaculate investment.

The “mating and ejaculation” males experienced a higher frequency of mitotic and meiotic divisions in the germline, as well as any costs associated with the maintenance of spermatozoa (Maklakov and Immler 2016) because they repeatedly ejaculate during mating. Poeciliid fish rarely renew non-used sperm reserves (Billard and Puissant 1969), so post-copulatory investment through dumping and/or reabsorbing unused sperm and production of new sperm is much lower than that associated with repeated ejaculation.

All focal males were anesthetized in an ice slurry prior to either the actual or sham removal of their gonopodium tip (see below). After surgery, the males were transferred to individual 7L tanks for 3 days. We then introduced a stimulus female and two rival males into each tank. The stimulus fish were rotated between tanks across treatments weekly to minimize any effect of female familiarity on male mating effort (Vega-Trejo et al. 2014) and to reduce variation in male dominance status among focal males (Harrison et al. 2023). Prior to the experiment, rival stimulus males were marked with a colored elastomer tag (Northwest Marine Technology, Shaw Island, WA) so that we could identify focal males (Aich et al. 2020a). Focal males were maintained in their respective treatments for 16 weeks, corresponding to the length of the breeding season in the wild population (Kahn et al. 2013). We collected data on focal males at the midpoint (week 8) and end (week 16) of the treatment period. We could then assess the effect of reproductive history (i.e., treatment), treatment duration, and their interaction on male attractiveness and male mate choice. In total, 17 of 180 males (naïve: *n* = 4; mating only: *n* = 6; mating and ejaculation: *n* = 7) died in the first 8 weeks, and 17 males (naïve: *n* = 8; mating only: *n* = 3; mating and ejaculation: *n* = 6) died in the second half of the experiment (i.e., weeks 8 to 16). There was no significant effect of treatment on survival (GLM with binomial error, week 0 to 8: χ²_2_ = 0.892; *P* = 0.640; week 8 to 16: χ²_2_ = 2.180; *P* = 0.336). The data from males that died during weeks 8 to 16 was included in the Week 8 analysis.

### Ablation surgery

After anesthesia in an ice slurry for 10 s, a focal male was placed on a glass slide and the gonopodium swung forward under a dissecting microscope. Males of the “mating only” treatment had their entire gonopodium tip removed with a blade (Diplomat Blades, Victoria, Australia), while the other males underwent the same procedure without ablation. Males did not regenerate the tip of their gonopodium (Supplementary Figure S1).

### Male attractiveness

**Figure 1:**
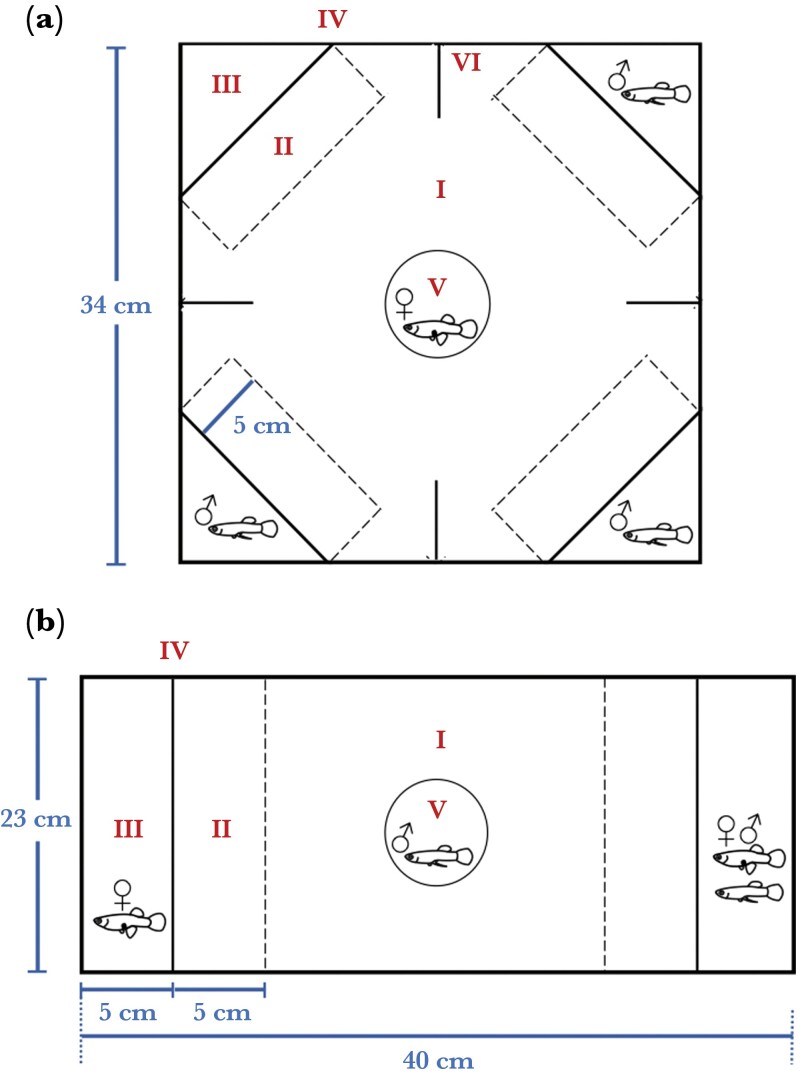
Upper views of experimental apparatus for (a) male attractiveness (four-choice trials) and (b) male mate choice (two-choice trials). Each apparatus contains (I) neutral zone; (II) association zones; (III) end sections; (IV) mesh barrier and mobile opaque screen; (V) plastic cylinder; (VI) black barrier. In the male attractiveness trials (a), a virgin female was housed within a plastic cylinder (V) in the neutral zone (I), and three of the four end sections (III) each contained one focal male. In the male mate choice trials (b), a focal male was kept within a plastic cylinder in the neutral zone, with a female (either with or without a male rival) placed in each of the end sections. After a 10-min acclimation period, we removed the opaque screen and the plastic cylinder to allow the focal fish to swim freely around the neutral and association zones (I and II). The time spent in each association zone was our measure of mate preference.

After 8 and 16 weeks, three males (one per treatment) were introduced into a test tank (34 cm × 34 cm × 8 cm). The tank had four corner compartments separated by mesh barriers and removable black screens (Figure 1a). Each male was randomly placed in one corner, with the fourth compartment left empty. Whether an assigned chamber was next to the empty corner did not alter the treatment effect (see Supplementary Material). A virgin female (26.24 ± 0.21 mm SL; *n* = 73) was placed in a Plexiglas cylinder in the center of the tank. After a 10-min acclimation period, the cylinder and black barriers were removed to allow the female to inspect and approach males for 10 min. All females were randomly selected from stock and swam actively during the trial (distance: 492–1397 cm). We recorded the time each female spent within 5 cm of each mesh barrier as an indication of male attractiveness. To justify the link between association time and mate choice in *G. holbrooki*, see Vega-Trejo et al. (2014) and a review (Dougherty 2020). To minimize human disturbance, a 5MP dome camera (CCTV Central, Victoria, Australia) was mounted above the tank, and the videos were analyzed blind to male treatment using Ethovision XT (Noldus Information Technology, Wageningen, Netherlands).

To control for any body size effect on male attractiveness, in each trial, we used three focal males of similar initial body size that completed their treatment at the same time (± 1 day). We conducted a total of 73 trials (Week 8: *n* = 39; Week 16: *n* = 34), with a mean size difference of 1.094 ± 0.009 mm SL.

### Male mate choice

To investigate the effect of reproductive history on male mate choice, we ran two-choice trials after 8 and 16 weeks. Focal males were given a choice between two size-matched females, one of whom was in the presence of a rival. The obvious prediction is that males will prefer the female that is on her own to reduce the risk of sperm competition or direct fighting for access to the female (e.g., Dosen and Montgomerie 2004; Bierbach et al. 2011). At the start of each trial, a focal male was introduced into a Plexiglas cylinder in the middle chamber of the test aquarium (40 cm × 23 cm × 10 cm) (Figure 1b). Two stock females (*n* = 173 pairs) with a size difference ranging from 0.001 to 2.698 mm SL (mean ± SE = 0.619 ± 0.005) were introduced into opposite end compartments. A stimulus stock male (22.234 ± 0.139 mm SL; *n* = 173) was placed randomly with one female. Stimulus females and males were wild caught and housed in single-sex stock tanks for at least 1 month before the trial. Following a 10-min acclimation period, the cylinder and the opaque screens separating the end compartments from the main chamber were removed. We then recorded the total distance the focal male swam and the time he spent in each association zone (<5 cm from the mesh barrier; Figure 1b) for 10 min using an overhead camera (as above). The video recordings were analyzed blind to male treatment in Ethovision XT to exclude experimenter bias. Each solitary female and female alongside a rival was used only once per treatment. They were alternately placed on the left and right sides of the tank between trials to prevent side biases.

### Statistical analysis

Our experimental and analysis plans were registered online before data collection (osf.io/swdv7). For each response variable (time females spent with each male; total distance males swam; total time males spent inspecting females; the proportion of time males spent with the solitary female), we used mixed models including male ID as a random factor, to account for repeated measurements of the same male. We included reproductive treatment (“naïve,” “mating only,” “mating and ejaculation”), treatment duration (8 weeks, 16 weeks), and their interaction in initial models. A nonsignificant interaction was removed from the final model to interpret the main effects (Engqvist 2005). Removing non-significant interactions did not significantly alter the model fit, as determined by log-likelihood ratio tests. Full model outputs (for both initial and final models) are provided in the Supplementary Material. Wald chi-square tests were performed to calculate the *P* value using the *Anova* function in the *car* package (R studio v1.3.1093 with R v4.0.5). We used type III sums of squares for models with the interaction term and type II sums of squares for models without the interaction. Results are presented as mean ± SE. The significance level is set at α = 0.05 (two tailed). We ran Tukey’s post hoc pairwise tests (*emmeans* package), if the treatment effect was significant. Any deviations from this approach are specified below.

#### Male attractiveness

We employed a two-step analysis to investigate male attractiveness. First, we tested whether females preferred to spend time with males rather than alone by comparing the proportion of time spent near the empty compartment to that expected by chance (= 0.25) using separate one sample *t*-tests for trials at weeks 8 and 16. The proportion of time was power-transformed to meet the assumption of normality (determined via Shapiro-Wilk tests). Second, we tested whether male reproductive history, treatment duration, and their interaction affected how much time a female spent with a male using a generalized linear mixed model (GLMM). Quasi-Poisson error was employed to ensure that the data variance conformed to the model assumption, which was assessed using a dispersion test in the *DHARMa* package. The total time females spent in the association zone of each male was the response variable, with female ID as a random factor to account for three males being tested in the same trial (Aich et al. 2020a).

#### Male mate choice

We ran linear mixed models to test the effects of male reproductive history, treatment duration, and their interaction on male: (1) distance swum and (2) time spent inspecting females. We analyzed the proportion of time males spent with the solitary female compared to the other female using a GLMM (binomial error, cbind function of absolute time with each female), where trial ID was considered as a random effect to account for overdispersion (Harrison 2014). Pair ID was included as a random factor to account for repeated usage of a combination of stimulus fish (max 3 trials per combination; one per treatment).

We further tested whether the proportion of time males of each type at 8 and 16 weeks, respectively spent with a solitary female differed from the null expectation (= 50%). To do this, we ran six separate intercept-only GLM models (quasi-binomial error, cbind function of absolute time with each female). An intercept of 0 corresponds to males spending 50% of the time with each female (ln(*p*/[1 − *p*]), where *p *= proportion of time with the solitary female). If the intercept is significantly greater than zero, this indicates that males spent significantly more time with the solitary female.

## RESULTS

### Male attractiveness

**Figure 2:**
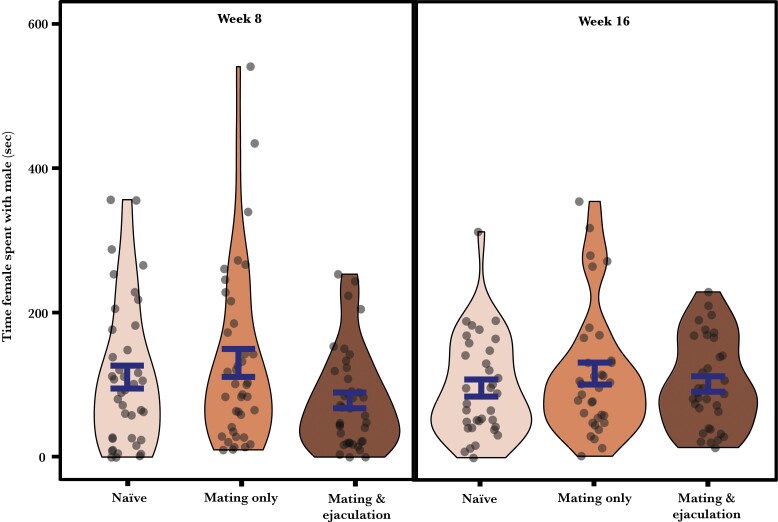
Effects of reproductive history and treatment duration on the time females spent with each male. Data are shown as mean ± SE, with the distribution of sample.

Test females spent 87 ± 3 % (Week 8) and 83 ± 2 % (Week 16) of their time in the association zones of compartments housing a male, which is significantly more than the 75% expected by chance (one-sample *t*-test, Week 8: *t*_38_ = 6.230; *P* < 0.001; Week 16: *t*_33_ = 4.079, *P* < 0.001). Reproductive history had no effect on the amount of time females spent with each type of male, indicating no effect on male attractiveness (χ²_2_ = 4.631; *P* = 0.099; Figure 2). There was also no effect of treatment duration on how much time females spent with males (χ²_1_ = 2.711; *P* = 0.100), nor any interaction of duration with reproductive history (χ²_2_ = 2.570; *P* = 0.277).

### Male mate choice

**Figure 3:**
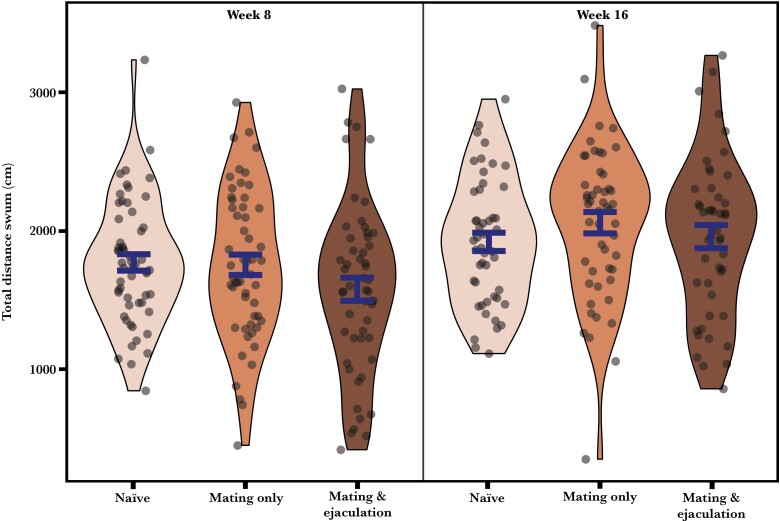
Total distance swum by males during mate choice trials. Sample distribution with mean and SE are shown.

**Figure 4:**
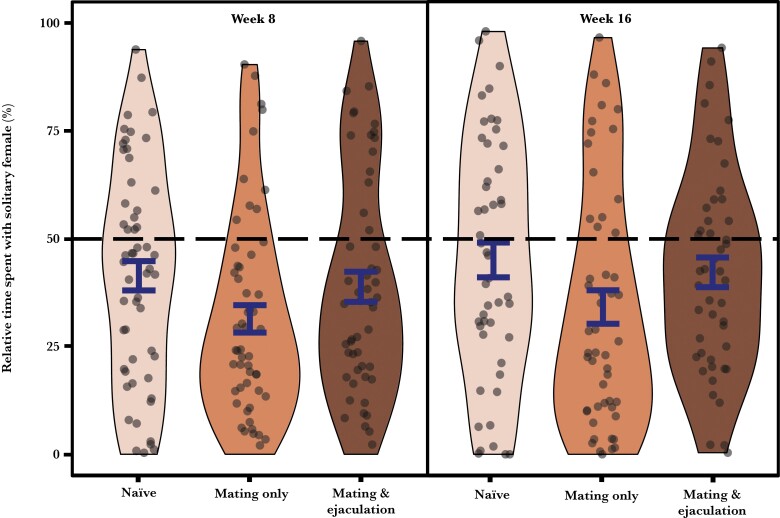
Effects of reproductive history and treatment duration on the proportion of time in the association zone that males spent with a female on her own. The sample distribution with mean and SE are shown.

Male reproductive history did not affect the total distance swum by males (χ²_2_ = 4.141; *P* = 0.126) (Figure 3), but it had a marginally significant effect on the total time spent inspecting females (χ²_2_ = 6.001; *P* = 0.0498), and the proportion of time spent with the solitary female (χ²_2_ = 6.017; *P* = 0.0494) (Figure 4). “Naïve” males spent more time than “mating and ejaculation” males with females (Tukey’s test, *P* = 0.045), but neither differed significantly from the time spent with females by “mating only” males (Tukey’s test, both *P* > 0.256). “Mating only” males spent less time than “naïve” or “mating and ejaculation” males with the solitary female, but the pairwise differences were not significant (Turkey’s test, *P* = 0.091 and 0.085). “Naïve” and “mating and ejaculation” males did not differ in the proportion of time spent with the solitary female (Turkey’s test, *P* = 0.999). Interestingly, mated males at both weeks 8 (GLM_mating only_ = −0.914 = 31%, *t*_53_ = −5.942, *P* < 0.001; GLM_mating and ejaculation_ = −0.376 = 39%, *t*_52_ = −2.267, *P* = 0.028) and weeks 16 (GLM_mating only_ = −0.699 = 34%, *t*_50_ = −3.637, *P* < 0.001; GLM_mating and ejaculation_ = −0.347 = 42%, *t*_46_ = −2.191, *P* = 0.034) as well as naïve male at weeks 8 (GLM = −0.396 = 41%, *t*_55_ =−2.547, *P* = 0.014) spent significantly *more* time associating with the female alongside a rival than with the solitary female. The only exception was naïve males at weeks 16 who did not show a preference (GLM = −0.195 = 45%, *t*_47_ =−1.056, *P* = 0.297).

Males swam more actively during trials conducted after 16 than 8 weeks (χ²_1_ = 22.720; *P* < 0.001) (Figure 3), but treatment duration did not affect the total inspection time (χ²_1_ = 3.599; *P* = 0.058) or the proportion of time spent with the solitary female (χ²_1_ = 0.461; *P* = 0.497)

Reproductive history and treatment duration did not interact to affect the total distance swum by males (χ²_2_ = 3.350; *P* = 0.187), the total time spent inspecting females (χ²_2_ = 2.644; *P* = 0.267), or the proportion of time spent with females that were alone (χ²_2_ = 0.112; *P* = 0.945).

## DISCUSSION

Reproductive history might affect male mate choice through accumulated experience that enhances the ability to assess the female quality and/or the social environment, hence the benefits and costs of choosiness (Anderson et al. 2007); or past reproductive effort may lower competitive ability, shifting a male’s mating preferences to females subject to less male–male competition (Venner et al. 2010; Pollo et al. 2022). Previous studies on *G. holbrooki* found that males with access to females grew slower and had a weaker immune response than naïve males (Iglesias-Carrasco et al. 2019). Mated males also produce fewer sperm than naïve males as they age (Aich et al. 2021). Chung et al. (2021) then separated the life-history effects of mating behavior and ejaculation. Past mating behavior imposed greater costs than repeated ejaculation on immunocompetence, growth, and future mating performance, while repeated ejaculation, but not past mating effort, reduced sperm production. In this study, we therefore predicted that mated males, especially “mating and ejaculation” males, would choose to associate with solitary females to avoid male-male fights and lower the level of sperm competition (Wong and McCarthy 2009). On the other hand, we expected females to prefer “naïve” males because they are in better condition. Alternatively, however, females might avoid “naïve” males to reduce costly sexual harassment (e.g., Pilastro et al. 2003; Agrillo et al. 2006; Langerhans 2011). For instance, female cockroaches that copulate with subordinate rather than dominant males benefit from extended lifespans (Moore et al. 2003). If this were the case, we might expect female *G. holbrooki* to avoid males in our female choice trials (i.e., spend significantly more time near the empty chamber).

### Male attractiveness

Females in our study spent more time associating with males than near the empty compartment, indicating that they did not actively avoid males, presumably because the benefits of receiving sperm and/or shoaling outweigh those of avoiding male sexual harassment. Females did not, however, discriminate between naïve and mated males. Similarly, Aich et al. (2020a) and Chung et al. (2021) found that male reproductive history, albeit excluding male–male encounters, did not affect male attractiveness. One explanation is that multiple mating reduces the need for female mate choice, particularly as there is no detectable difference in sperm quality or its fertilization success between naïve and mated males (Aich et al. 2021; Chung et al. 2021). However, daughters sired by mated males mature much later than those sired by naïve males (Aich et al. 2020b), implying a fitness cost for females.

### Male mate choice

We found a significant effect of reproductive history on the total time males spent inspecting females. “Naïve” males spent significantly more time than “mating and ejaculation” males inspecting females. This finding is consistent with the general claim that males who have invested heavily into reproduction have fewer resources to allocate to future mating attempts (Lemaître et al. 2015; Hooper et al. 2017). Importantly, our study revealed that the investment into past mating behavior or ejaculation *alone* did not affect the total time spent near females, as there was no significant difference in inspection time between “naïve” and “mating only” males, or between “mating only” and “mating and ejaculation” males.

### Why do males associate?

Contrary to expectations (e.g., Wong and McCarthy 2009), males from five of the six treatment-by-age at testing combinations spent significantly less time with solitary females than females with a rival male nearby. There was, however, no effect of male reproductive history on the strength of male choice, implying that the costs of past reproductive effort did not affect a male’s ability to discriminate between potential mates. Our finding that males tended to associate with each other when seeking a potential mate requires an explanation. All else being equal, choosing a female who is already near another male should increase the likelihood of shared paternity. Interestingly, Callander et al. (2012) observed that male *G. holbrooki* did not avoid competitors when choosing which of two females to associate with. Specifically, in a two-choice trial where two males were placed together to choose between a large and a small female (8 mm SL difference), both males associated with the larger female instead of avoiding each other. This finding could, however, have been due to males preferring larger females irrespective of the presence of rivals. In our current study, we confirmed the existence of a male–male association bias even when both females are the same size.

Several mechanisms might drive male–male association in mosquitofish. First, males might use other males as a cue to more efficiently identify prospective mates. For example, male wolf spiders and fiddler crabs use changes in courtship by other males to inform them of the nearby presence of females and then adjust their courtship accordingly (Milner et al. 2010; Clark et al. 2012). Following the same reasoning, male mosquitofish may decrease the effort put into mate searching by utilizing information that reveals the location of potential mates (e.g., harassment behavior of other males consistent with the presence of a female). Second, when two males approach a female together, this might increase the likelihood of successful insemination by lowering the female’s ability to evade mating attempts. However, it is worth noting that male–male interference might instead reduce the number of successful mating attempts (Pilastro et al. 2003). To better determine if male–male association during mate choice is adaptive for this reason, future studies should compare the number of successful inseminations per male by males on their own versus males in a pair or group.

Third, the presence of a male near a female may provide useful social information (Danchin et al. 2004; Lee et al. 2016). For instance, males may copy the mate choice of other males to reduce their decision time and uncertainty during mate assessment (Schlupp and Ryan 1997; Plath et al. 2008; Vakirtzis 2011; Auld and Godin 2015). Mate copying is common for sexually inexperienced individuals (meta-analysis: Jones and DuVal 2019); however, in our study, naïve males did not exhibit a stronger preference than experienced males for females with another male. Moreover, physical contact between the model male and a female in other poeciliids (*Poecilia mexicana*: Ziege et al. 2009; Bierbach et al. 2011; *P. reticulata*: Dosen and Montgomerie 2004; Makowicz et al. 2010) usually reduces male mate choice copying, which is contrary to our findings. Fourth, males might prefer females in the vicinity of a rival because of a preference for (1) more active female if being near another male alters her behavior (e.g., Dadda 2015), (2) an area that contains more conspecifics as a cue about habitat quality or resource abundance (i.e., conspecific attraction; Stamps 1988; Reed and Dobson 1993; Ahlering et al. 2010), or (3) simply due to benefits of shoaling (Herbert-Read et al. 2011; Pazmino et al. 2020). Researchers could conduct two-choice trials with the appropriate designs to distinguish among these possibilities. For instance, if males prefer a female near a rival due to her behavioral change, a male should still prefer such a female even if the rival is not visible to the test male (e.g., Wong and McCarthy 2009).

## CONCLUSION

Neither past mating behavior, ejaculation, nor their combined effects influenced male attractiveness in *G. holbrooki*. Similarly, we found no effect of the differences in treatment duration for male reproductive history on his attractiveness. Likewise, there were no clear effects of a male’s reproductive history on his mate choice. We did, however, observe an unusual tendency for males to prefer females in the vicinity of a rival over a solitary female. This preference was stronger, albeit not significantly so, in males that had previously invested into mating effort. Much is known about how female mate choice is influenced by her mating history (Jones and DuVal 2019; Richardson and Zuk 2023), but almost nothing is known about how a male’s mating history affects his mate choice. Here, we reveal the relative contributions of past mating behavior and ejaculation, and variation in the duration of this investment to male mate choice. This study is a first step in better understanding how individual components of reproductive history affect male mate choice in a species with intense sperm competition.

## Supplementary Material

arae002_suppl_Supplementary_Material

## Data Availability

Analyses reported in this article can be reproduced using the data and code provided by Chung et al. (2024).
