# Supplementary material for: Effects of past mating behavior versus past ejaculation on male mate choice and male attractiveness
Source: Behav Ecol. 2024 Jan 17;35(2):arae002. doi: 10.1093/beheco/arae002 (PMC10807976; doi:10.1093/beheco/arae002)
Supplement: arae002_suppl_Supplementary_Material [file arae002_suppl_supplementary_material.docx]

Supplementary material

**Effects of past mating behavior versus past ejaculation on male mate choice and male attractiveness**

**Part 1. Ablation surgery**

**
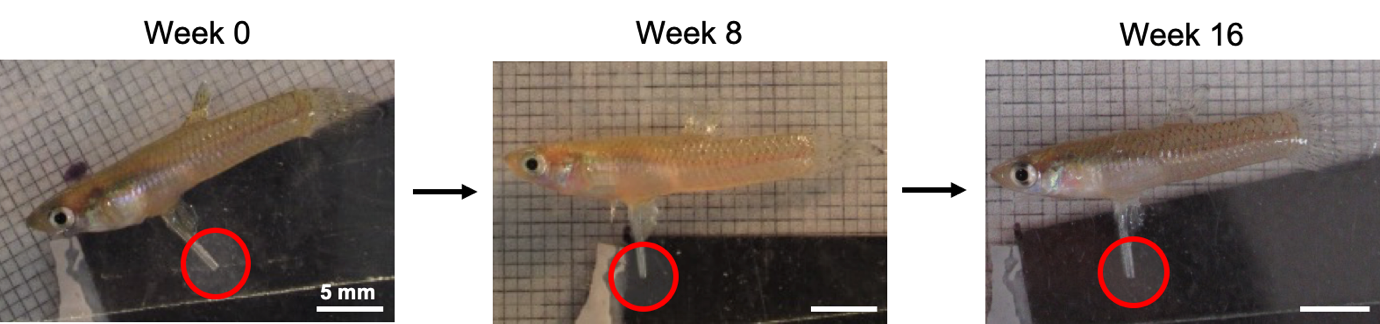
**

**Figure S1.** Photographs demonstrating that ablated males did not regenerate the tip of their gonopodium throughout the experimental period.

**Part 2. Statistical outputs from models**

1. **Time females spent with each male (i.e., male attractiveness)**
2. Initial model including the interaction between reproductive history and treatment duration

|  | | | | Estimate | *SE* | | *χ²* (df) | *P* |
| --- | --- | --- | --- | --- | --- | --- | --- | --- |
| Intercept (Week 16, Naïve) | | | | 4.640 | 0.133 | | 1225.767 (1) | **<0.001** |
| Treatment duration (Week 8) | | | | -0.131 | 0.180 | | 0.528 (1) | 0.468 |
| Reproductive history (Mating only) | | | | 0.165 | 0.176 | | 0.988 (2) | 0.610 |
| Reproductive history (Mating & ejaculation) | | | | 0.137 | 0.176 | |  |  |
| Treatment duration (Week 8) * Reproductive history (Mating only) | | | | 0.126 | 0.244 | | 2.570 (2) | 0.277 |
| Treatment duration (Week 8) * Reproductive history (Mating & ejaculation) | | | | -0.259 | 0.253 | |  |  |
| **Random effect** | Variance | *SD* | Number of groups | | |  |  |  |
| Female ID | <0.001 | <0.001 | 73 | | |  |  |  |
| Male ID | <0.001 | <0.001 | 125 | | |  |  |  |

1. Final model excluding the non-significant interaction

|  | | | | Estimate | *SE* | | *χ²* (df) | *P* |
| --- | --- | --- | --- | --- | --- | --- | --- | --- |
| Intercept (Week 16, Naïve) | | | | 4.662 | 0.108 | |  |  |
| Treatment duration (Week 8) | | | | -0.166 | 0.101 | | 2.711 (1) | 0.100 |
| Reproductive history (Mating only) | | | | 0.228 | 0.122 | | 4.631 (2) | 0.099 |
| Reproductive history (Mating & ejaculation) | | | | 0.007 | 0.127 | |  |  |
| **Random effect** | Variance | *SD* | Number of groups | | |  |  |  |
| Female ID | <0.001 | <0.001 | 73 | | |  |  |  |
| Male ID | <0.001 | <0.001 | 125 | | |  |  |  |

1. Exclusion of the non-significant interaction did not significantly reduce model fit in the final model

|  | df | AIC | BIC | Log-likelihood | Deviance | χ²_2_ | *P* |
| --- | --- | --- | --- | --- | --- | --- | --- |
| Initial model (a) | 9 | 2484.5 | 2515.0 | -1233.2 | 2466.5 | 2.565 | 0.277 |
| Final model (b) | 7 | 2483.1 | 2506.8 | -1234.5 | 2469.1 |  |  |

1. Additional analysis showing no effect of a male’s chamber location on the time females spent with him

|  | | | | Estimate | *SE* | | *χ²* (df) | *P* |
| --- | --- | --- | --- | --- | --- | --- | --- | --- |
| Intercept (Week 16, not middle, Naïve) | | | | 4.600 | 0.114 | |  |  |
| Treatment duration (Week 8) | | | | -0.159 | 0.100 | | 2.505 (1) | 0.114 |
| Chamber location (middle) | | | | 0.181 | 0.105 | | 2.985 (1) | 0.084 |
| Reproductive history (Mating only) | | | | 0.225 | 0.122 | | 4.949 (2) | 0.084 |
| Reproductive history (Mating & ejaculation) | | | | -0.010 | 0.127 | |  |  |
| **Random effect** | Variance | *SD* | Number of groups | | |  |  |  |
| Female ID | <0.001 | <0.001 | 73 | | |  |  |  |
| Male ID | <0.001 | <0.001 | 125 | | |  |  |  |

1. **Male mate choice**

**2-1 Total distance swum by males**

1. Initial model including the interaction between reproductive history and treatment duration

|  | | | | Estimate | *SE* | | *χ²* (df) | *P* |
| --- | --- | --- | --- | --- | --- | --- | --- | --- |
| Intercept (Week 16, Naïve) | | | | 1938.431 | 76.574 | | 640.836 (1) | **<0.001** |
| Treatment duration (Week 8) | | | | -142.993 | 93.624 | | 2.333 (1) | 0.127 |
| Reproductive history (Mating only) | | | | 145.907 | 103.283 | | 2.507 (2) | 0.286 |
| Reproductive history (Mating & ejaculation) | | | | 9.239 | 105.602 | |  |  |
| Treatment duration (Week 8) * Reproductive history (Mating only) | | | | -177.880 | 126.348 | | 3.350 (2) | 0.187 |
| Treatment duration (Week 8) * Reproductive history (Mating & ejaculation) | | | | -219.883 | 128.181 | |  |  |
| **Random effect** | Variance | *SD* | Number of groups | | |  |  |  |
| Female pair ID | 34046 | 184.5 | 173 | | |  |  |  |
| Male ID | 60693 | 246.4 | 163 | | |  |  |  |
| Residual | 190647 | 436.6 |  | | |  |  |  |

1. Final model excluding the non-significant interaction

|  | | | | Estimate | *SE* | | *χ²* (df) | *P* |
| --- | --- | --- | --- | --- | --- | --- | --- | --- |
| Intercept (Week 16, Naïve) | | | | 2009.130 | 65.260 | |  |  |
| Treatment duration (Week 8) | | | | -275.450 | 57.780 | | 22.720 (1) | **<0.001** |
| Reproductive history (Mating only) | | | | 51.010 | 79.140 | | 4.141 (2) | 0.126 |
| Reproductive history (Mating & ejaculation) | | | | -108.600 | 79.850 | |  |  |
| **Random effect** | Variance | *SD* | Number of groups | | |  |  |  |
| Female pair ID | 29509 | 171.8 | 173 | | |  |  |  |
| Male ID | 60879 | 246.7 | 163 | | |  |  |  |
| Residual | 195643 | 442.3 |  | | |  |  |  |

1. Exclusion of the non-significant interaction did not significantly reduce model fit in the final model

|  | No. parameter | AIC | BIC | Log-likelihood | Deviance | χ²_2_ | *P* |
| --- | --- | --- | --- | --- | --- | --- | --- |
| Initial model (a) | 9 | 4761.1 | 4794.7 | -2371.6 | 4743.1 | 3.328 | 0.189 |
| Final model (b) | 7 | 4760.5 | 4786.6 | -2373.2 | 4746.5 |  |  |

- 1. **Total amount of time male spent inspecting two females**

1. Initial model including the interaction between reproductive history and treatment duration

|  | | | | Estimate | *SE* | | *χ²* (df) | *P* |
| --- | --- | --- | --- | --- | --- | --- | --- | --- |
| Intercept (Week 16, Naïve) | | | | 283.857 | 16.619 | | 291.519 (1) | **<0.001** |
| Treatment duration (Week 8) | | | | -2.443 | 19.647 | | 0.014 (1) | 0.905 |
| Reproductive history (Mating only) | | | | -33.684 | 22.665 | | 7.916 (2) | **0.019** |
| Reproductive history (Mating & ejaculation) | | | | -65.099 | 23.145 | |  |  |
| Treatment duration (Week 8) * Reproductive history (Mating only) | | | | 35.236 | 26.779 | | 2.644 (2) | 0.267 |
| Treatment duration (Week 8) * Reproductive history (Mating & ejaculation) | | | | 40.380 | 27.185 | |  |  |
| **Random effect** | Variance | *SD* | Number of groups | | |  |  |  |
| Female pair ID | 1121 | 33.49 | 173 | | |  |  |  |
| Male ID | 3635 | 60.29 | 163 | | |  |  |  |
| Residual | 8693 | 93.24 |  | | |  |  |  |

1. Final model excluding the non-significant interaction

|  | | | | Estimate | *SE* | | *χ²* (df) | *P* |
| --- | --- | --- | --- | --- | --- | --- | --- | --- |
| Intercept (Week 16, Naïve) | | | | 270.040 | 14.300 | |  |  |
| Treatment duration (Week 8) | | | | 22.710 | 12.010 | | 3.599 (1) | 0.058 |
| Reproductive history (Mating only) | | | | -14.700 | 17.680 | | 6.001 (2) | **0.0498** |
| Reproductive history (Mating & ejaculation) | | | | -43.070 | 17.830 | |  |  |
| **Random effect** | Variance | *SD* | Number of groups | | |  |  |  |
| Female ID | 1157 | 34.01 | 173 | | |  |  |  |
| Male ID | 3687 | 60.72 | 163 | | |  |  |  |
| Residual | 8649 | 93.00 |  | | |  |  |  |

1. Exclusion of the non-significant interaction did not significantly reduce model fit in the final model

|  | No. parameter | AIC | BIC | Log-likelihood | Deviance | χ²_2_ | *P* |
| --- | --- | --- | --- | --- | --- | --- | --- |
| Initial model (a) | 9 | 3814.0 | 3847.6 | -1898.0 | 3796.0 | 2.684 | 0.261 |
| Final model (b) | 7 | 3812.6 | 3838.8 | -1899.3 | 3798.6 |  |  |

- 1. **Proportion of time males spent with the solitary female**

1. Initial model including the interaction between reproductive history and treatment duration

|  | | | | Estimate | *SE* | | *χ²* (df) | *P* |
| --- | --- | --- | --- | --- | --- | --- | --- | --- |
| Intercept (Week 16, Naïve) | | | | -0.484 | 0.243 | | 3.961 (1) | **0.047** |
| Treatment duration (Week 8) | | | | -0.160 | 0.331 | | 0.233 (1) | 0.630 |
| Reproductive history (Mating only) | | | | -0.538 | 0.333 | | 3.724 (2) | 0.155 |
| Reproductive history (Mating & ejaculation) | | | | 0.032 | 0.339 | |  |  |
| Treatment duration (Week 8) * Reproductive history (Mating only) | | | | 0.110 | 0.459 | | 0.112 (2) | 0.945 |
| Treatment duration (Week 8) * Reproductive history (Mating & ejaculation) | | | | -0.039 | 0.463 | |  |  |
| **Random effect** | Variance | *SD* | Number of groups | | |  |  |  |
| Female pair ID | 0.200 | 0.447 | 173 | | |  |  |  |
| Male ID | <0.001 | <0.001 | 163 | | |  |  |  |
| Trial ID | 2.576 | 1.605 | 309 | | |  |  |  |

1. Final model excluding the non-significant interaction

|  | | | | Estimate | *SE* | | *χ²* (df) | *P* |
| --- | --- | --- | --- | --- | --- | --- | --- | --- |
| Intercept (Week 16, Naïve) | | | | -0.497 | 0.197 | |  |  |
| Treatment duration (Week 8) | | | | -0.135 | 0.199 | | 0.461 (1) | 0.497 |
| Reproductive history (Mating only) | | | | -0.481 | 0.228 | | 6.017 (2) | **0.0494** |
| Reproductive history (Mating & ejaculation) | | | | 0.011 | 0.231 | |  |  |
| **Random effect** | Variance | *SD* | Number of groups | | |  |  |  |
| Female pair ID | 0.204 | 0.452 | 173 | | |  |  |  |
| Male ID | <0.001 | <0.001 | 163 | | |  |  |  |
| Trial ID | 2.574 | 1.604 | 309 | | |  |  |  |

1. Exclusion of the non-significant interaction did not significantly reduce model fit in the final model

|  | Df | AIC | BIC | Log-likelihood | Deviance | χ²_2_ | *P* |
| --- | --- | --- | --- | --- | --- | --- | --- |
| Initial model (a) | 9 | 3361.4 | 3395.0 | -1671.7 | 3343.4 | 0.112 | 0.945 |
| Final model (b) | 7 | 3357.5 | 3383.7 | -1671.8 | 3343.5 |  |  |
